# Influence of state reopening policies in COVID-19 mortality

**DOI:** 10.1038/s41598-022-05286-9

**Published:** 2022-01-31

**Authors:** Ka-Ming Tam, Nicholas Walker, Juana Moreno

**Affiliations:** 1 Department of Physics and Astronomy, Louisiana State University, Baton Rouge, LA 70803 USA; 2 Center for Computation and Technology, Louisiana State University, Baton Rouge, LA 70803 USA

**Keywords:** Computational models, Epidemiology, Population dynamics

## Abstract

By the end of May 2020, all states in the US have eased their COVID-19 mitigation measures. Different states adopted markedly different policies and timing for reopening. An important question remains in how the relaxation of mitigation measures is related to the number of casualties. To address this question, we compare the actual data to a hypothetical case in which the mitigation measures are left intact using a projection of the data from before mitigation measures were eased. We find that different states have shown significant differences between the actual number of deaths and the projected figures within the present model. We relate these differences to the states different policies and reopening schedules. Our study provides a gauge for the effectiveness of the approaches by different state governments and can serve as a guide for implementing best policies in the future. According to the Pearson correlation coefficients we obtained, the face mask mandate has the strongest correlation with the death count than any other policies we considered.

## Introduction

At of the beginning of November, there are close to 10 million confirmed cases and more than 237,000 casualties attributed to COVID-19 in the United States. Although the first case was confirmed on January 20, the number of reported cases was rather low until early March. The number of reported cases then dramatically increased in early March.

Since the growth rate of infections was alarmingly high in most areas by mid-March, the majority of states implemented mitigation efforts by the end of March. These measures included closing restaurants and bars, shutting down schools, and implementing stay-at-home orders. By early April, nearly all states were under some form of lock-down. The infections tapered down appreciably due to these mitigation efforts. The daily number of new cases seemed to peak in early April and both the number of new cases and deaths steadily decreased from April to late May^1^.

While the total number of infections never dropped below 16,000 per day, many states decided to reduce their mitigation efforts after this first wave^1^. By the end of May, all states had rolled back their restrictions in some capacity. Certain states were more aggressive in reopening than others. While the number of infections was not particularly large at the beginning of reopening since the observed effect of the spread is delayed due to the incubation time and waiting time to receive testing results, many states saw a sharp increase in the number of cases between late May and early June^1–5^. This concerning situation forced several states to scale back plans for reopening in order to stabilize the exponentially increasing number of cases^6^.

There are several factors which are expected to reduce COVID-19 mortality rate. Adequate testing was rather limited before May. Testing has since expanded appreciably and stabilized at about 800,000 per day^1^. One would expect an increase in testing rate to reduce the number of fatalities, as people who tested positive as well as their close contacts should be quarantined. Moreover, the death rate was particularly high at the beginning of the pandemic due to the large number of infections in high risk groups, such as residents of assisted living facilities. Finally, improvement in treatments should also help to reduce the mortality rate. Together with the awareness of preventative measures, such as wearing masks in public areas, all of these factors should help to reduce the mortality rate and the number of casualties per day providing that the mitigation efforts remain intact.

Since all of these factors point to a decrease in the number of fatalities, the marked increase in the number of deaths since late May clearly indicates that relaxation of the mitigation efforts is the prominent reason for the increase. As we find in the present study, states which were more aggressive in reopening, in terms of both the timing and the restrictions lifting, have experienced a higher percentage increase in death counts. Therefore we believe understanding the relation between reopening policies and death count is a timely topic.

This paper provides an estimate of the change in the number of fatalities with respect to easing mitigation efforts. We not only quantify the additional number of deaths, but more importantly, by comparing between different states, this study provides clues to the reopening strategy which could minimize loss of life. We find significant differences between different states, as while most states display an increase in the number of fatalities, a handful of states actually show a decrease. This should be important and timely information as another infection wave may occur in the winter season. Some recent articles which study the effects of the reopening can be found in Refs.^7–9^.

## Model

The standard Susceptible-Infected-Recovered (SIR) model^10,11^ is modified to consider the number of quarantined people. Related models have been considered for the simulation of the spreading of the COVID-19^12–74^. This model is essentially a mean field approach that coarse-grains the entire population into different categories according to their viral infection status. The interactions between different categories are governed by the differential equations we describe below.

The coarse-grained mean field approach allows the simulation to proceed without adjusting many of the tunable parameters. This is often not the case for a generic fitting of a time series. While the flexibility of the present model may not be able to predict details at the local level (a city or a county) over short durations (a few days) which obviously requires more parameters for modelling, the present scheme can provide a trend for large areas (a state or a country) over longer durations (a few weeks or months). The main goal of the present study is to investigate the effects of state-wise reopening in the summer months, which the present model should properly address. We refer the readers to recent publications^75,76^ for more detailed discussion of the limitations of the present model for simulating the COVID-19 pandemic.

The dynamics of the model are governed by the following equations:^12,13,75,76^

1 $$\frac{dS(t)}{dt} = -\beta \frac{S(t)I(t)}{N},$$

2 $$\frac{dI(t)}{dt} = \beta \frac{S(t)I(t)}{N} -(\alpha +\eta )I(t),$$

3 $$\frac{dQ(t)}{dt} = \eta I(t)-\delta (t) Q(t) - \xi (t) Q(t),$$

4 $$\frac{dR(t)}{dt} = \xi (t) Q(t) +\alpha I(t),$$

5 $$\frac{dC(t)}{dt} = \delta (t) Q(t),$$

where *N* is the total population under consideration, *S* is the population susceptible to infection, *I* is the population that is not identified as able to transmit the virus to others, *Q* is the population of identified positive cases which are quarantined, *R* is the population of recovered patients (including both previously identified and unidentified cases), and *C* is the number of casualties.

The following parameters characterize the model: $\beta$ is the infection rate, $\eta$ is the detection rate of infected patients, $\alpha$ is the recovery rate of asymptomatic infected people, $\xi$ is the recovery rate of the quarantined patients, and $\delta$ is the casualty rate of the quarantined. All of the parameters are in units of (1/day). The quarantined population *Q* is composed of the identified positive cases regardless of whether they are hospitalized or at home. Presymptomatic case is not considered separately.

We further assume that all casualties had been in quarantine prior to death and we consider that only $\xi$ and $\delta$ are time dependent out of the coefficients. All of these assumptions are approximations made to allow for inference of the model parameters from the currently available data.

The total death count at time *t*, *D*(*t*), can be estimated as:

6 $$D(t) = \int _{0}^{t} \frac{C(\tau )}{d\tau } d\tau.$$

The confirmed positive count is $P(t)= Q(t)+R_Q(t)+C(t)$, where $R_Q(t)$ are the recovered patients previously in quarantine. *P*(*t*) can be estimated as:

7 $$\begin{aligned} P(t)= & {} \int _{0}^{t} \frac{dP(\tau )}{d\tau } d \tau \end{aligned}$$

8 $$\begin{aligned}= & {} \int _{0}^{t} \left( \frac{dQ(\tau )}{d\tau } +\frac{dR_Q(\tau )}{d\tau }+\frac{dC(\tau )}{d\tau }\right) d\tau \nonumber \\= & {} \int _{0}^{t} \eta I(\tau ) d{\tau }. \end{aligned}$$

## Method

The method for modeling the effect of the mitigation efforts is done by considering two sets of parameters, one before the stay-at-home order is in place and the other after the social distancing measures are in place, as discussed in our previous work^75^.

The median time between infection and the onset of symptoms, and the median time between the onset of symptoms and death are five and eight days, respectively^2–5^. We only use the mean value, however the distribution is not self-averaging^2–5^. This also leads to a major difficulty on calculating the “confidence level”. For a very limited amount of data following an unknown distribution, it is rather difficult to provide a realistic estimate of the confidence interval^77^. A more sophisticated model is needed to take such effects into consideration. Within the current approach we estimate the mortality rate to be $\displaystyle \delta _0 \approx 0.023 / (5+8) \approx 0.0018/$day, where the accumulated mortality rate is about 2.3%^78^. The recovery rate of asymptomatic people, $\alpha$, is estimated based on the average time to recovery or death from infection, which are both thirteen days, and that approximate half of the infected people are asymptomatic^79^. Then, we estimate $\alpha =0.5/13 \approx 0.0385/$day. Prior to the availability of fourteen days of data the mortality rate, $\delta _0$, is estimated by combining the accumulated mortality rate data and the median time between infection and death. After fourteen days of data are available, we estimate both the death rate, $\delta (t)$, and the recovery rate of the quarantined, $\xi (t)$, from the raw data, using the assumption that the average time from the onset of symptoms to death or recovery is eight days. The percentage of asymptomatic people remains fixed to 50%^79^.

Instead of extrapolating the data from other areas or countries, we choose to determine it from the casualty and confirmed case counts in each state separately. We estimate the first set of parameters by fitting the data at the beginning of the epidemic to an exponential growth curve. We consider the effects of social distancing measures to be reflected in two parameters, the reduction of the infection rate and the first day when the measurements are effective since there is a time delay in the influence the stay-at-home orders have on the number of cases and deaths. We determine both parameters by minimizing the $\chi ^2$ of the values and daily changes of the deaths and the confirmed infected counts based on data from before April 30th^76^. The data for the daily casualties and new cases are obtained from the database of the New York Times^80^.

The major difficulty on modelling the spreading of COVID-19 is the influence of state or country policies^81^. Policies such as reopening of different categories of venues or mask mandate cannot be factored into the model prior to the state government announcement. On the other hand, modelling without the external perturbations from the changes in policies can be used as a posterior gauge to investigate the effects due to the changes in policies.

The focus of the present study is to employ the data before May 2020 to model the spreading of COVID-19. Before May 2020, most of the mitigation efforts were not relaxed. The data from the model should present the dynamics of COVID-19 without the relaxation of the mitigation efforts. We then compared the projected death counts from modeling without policies changes factored in to the actual number of death counts. This ratio of the actual death counts to the projected death counts should then represent a measure of the effects from the changes in policy. We then find the correlation between this ratio and the reopening dates of different categories of venues, and also the mask mandate to find out the policy with the large correlation to the ratio between actual and projected death counts.

## Results

### Change in the mortality from reopening

Within the present model, there are two major routes for slowing the initial exponential growth of the epidemic, which is characterized by the parameter $\beta -(\alpha + \eta )$. The first route is to decrease the infection rate $\beta$ and the second route is to increase the testing rate $\eta$. Mitigation efforts in the spring sharply reduced the infection rate^76^. Testing rate also grew during the spring and summer^1^. Increasing the recovery rate of unidentified infected people, $\alpha$, can also reduce the spread, but this is more difficult to achieve. Overall, we can expect that the recovery rate should improve slightly over time. The average age of an infected person has trended towards decreasing since March^1^. This fact should lower the death rate among infected cases, since our model does not differentiate between different age groups.

**Figure 1:**
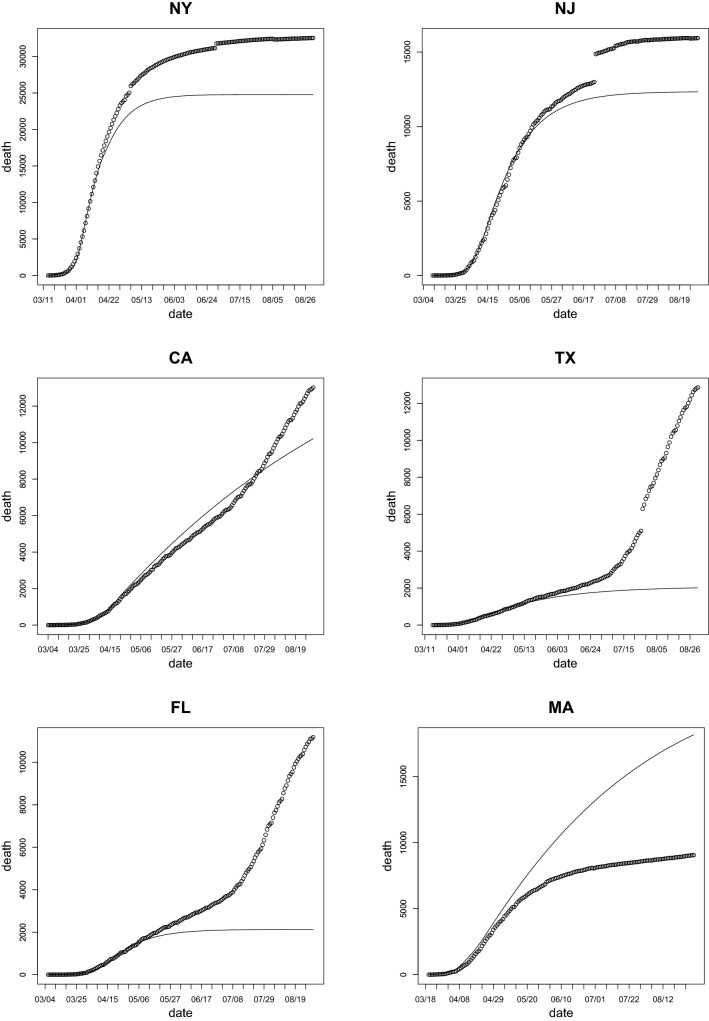
Total death count (dots)^80^ and projected death count without easing the mitigation (solid line) as functions of time for the states of New York, New Jersey, California, Texas, Florida, and Massachusetts using estimate by the data before the end of April.

**Figure 2:**
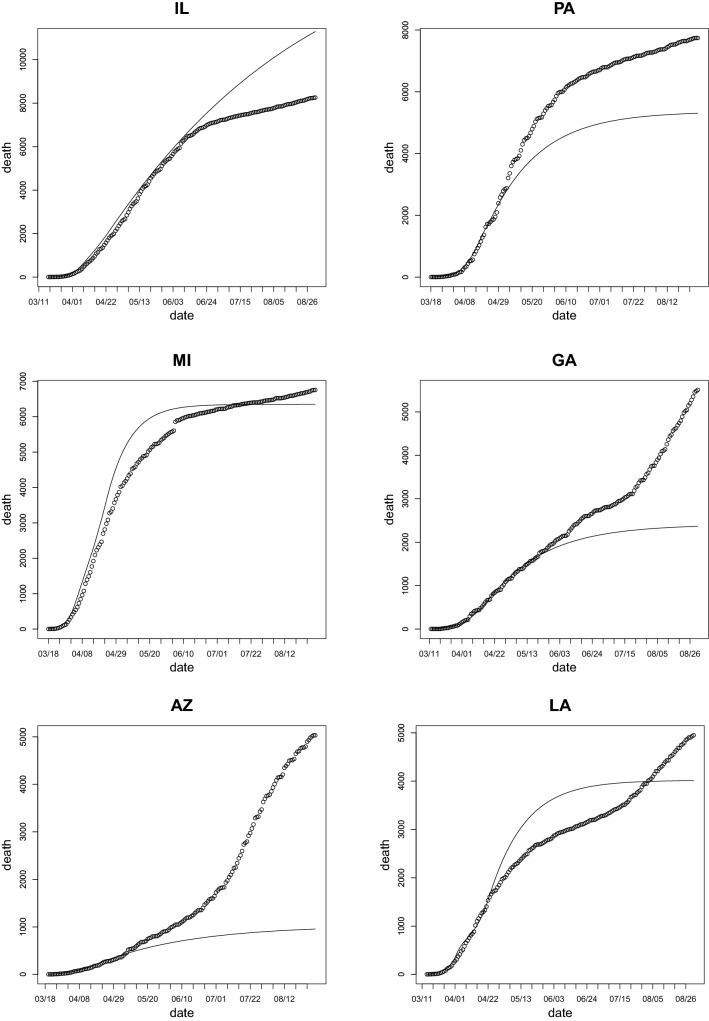
Total death count (dots)^80^ and projected fatalities count without easing the mitigation efforts (solid line) as functions of time for the states of Illinois, Pennsylvania, Michigan, Georgia, Arizona, and Louisiana using estimate by the data before the end of April.

All of these factors point out that the major, if not the only, reason for drastic increases on the number of infections and deaths is the relaxation of the mitigation efforts and by consequence an increase in the infection rate. If the mitigation efforts were to have remained intact, one would naively expect that both the number of infections and fatalities would decrease over time.

We attempt to quantify how many additional people died due to the relaxation of mitigation efforts. We project the number of casualties under the assumption that the mitigation efforts remain intact from the end of April to the end of August, and compare this projection with the actual death counts by the end of August. We choose this date for the reason that the summer peak seems to be passed by the end of August.

**Figure 3:**
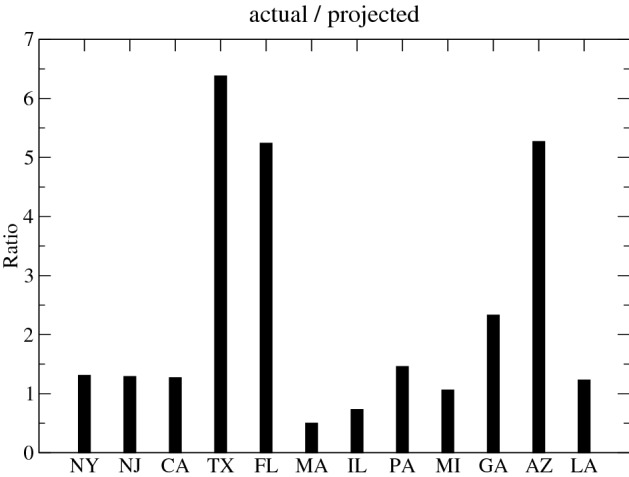
Bar chart with the ratio between the actual and the projected death count by the end of August for the twelve states with the largest counts. The projected count is calculated assuming that the mitigation efforts have been maintained since the end of April. This projection represents the ideal upper bound for the number of casualties as other factors which should lower the death count are not taken into account in the present model. See the text for details. Most states are within 50% of the projection. Two states, Massachusetts and Illinois, demonstrate a lower death number relative to the projections. Three states, Texas, Florida, and Arizona, show a substantial increase relative to the projections. Another state which shows an increase greater than a factor of two is Georgia. Notes: The data can be found in Table 1.

*We investigate the twelve states with the largest number of casualties by the end of August* They are New York, New Jersey, California, Texas, Florida, Massachusetts, Illinois, Pennsylvania, Michigan, Georgia, Arizona, and Louisiana. Figures 1 and 2 display the projected number of deaths, assuming that the mitigation efforts and all of the parameters in the model remain unchanged since the end of April, alongside the actual number of deaths. Table 1 displays the confirmed actual and the projected number of deaths by August 31st, as well as the ratio between both counts ($R_{death}$). Figure 3 shows the ratio between the actual and the projected death count by the end of August as a bar chart.

From the data presented in Figs. 1, 2 and Table 1, one can conclude that there are marked differences in the increase of the death count among various states. As we discussed above, the major factor in this difference should be due to the distinct policies in relaxing the mitigation efforts. An interesting and important topic is to find any possible correlation between these policies and the increase or decrease of fatalities.

**Table 1:** The actual confirmed and the projected number of deaths by August 31st. The projected count is obtained by assuming the mitigation efforts in place at the end of April were maintained by the end of August. The last column is the factor of increase (if larger than 1) or decrease (if smaller than 1) of the actual death count when compared with the projection.

State	Actual	Projected	Actual/projected
New York	32,541	24,788	1.31
New Jersey	15,945	12,333	1.29
California	13,020	10,214	1.27
Texas	12,857	2,015	6.38
Florida	11,186	2,133	5.24
Massachusetts	9,060	18,184	0.50
Illinois	8,258	11,296	0.73
Pennsylvania	7743	5304	1.46
Michigan	6756	6348	1.06
Georgia	5506	2365	2.33
Arizona	5031	955	5.27
Louisiana	4950	4011	1.23

### Reopening schedules of different states

In this section, we break down the details of the reopening policies of various states by considering the status of five categories of activities. These include the reopening of outdoor restaurants, indoor restaurants, bars, gyms, and stores. We emphasize that the reopening dates of different categories of venues are not a reflection for the dates for which the effects become measurable.

The timings for reopening these businesses vary significantly between states. For example, indoor restaurants were allowed to open on April 27th in Georgia, but not until September 1st in New Jersey. Besides the schedule of reopening of different businesses, the face mask mandate can also be an important policy which affects the spreading of the virus^82^, therefore we also considered the dates face mask mandates were enacted. The schedule for reopening and the date for the state mask mandate, if applicable, are displayed in Table 2.

**Table 2:** The reopening policies of different states. These include the dates of reopening of outdoor restaurants, indoor restaurants, bars, gyms, and stores. The date of the state mask mandate is also listed in the last column. An entry of “NA” indicates that a statewide reopening of the particular category or mask mandate has not been implemented by the September 1st^6^.

State	Outdoor restaurants	Indoor restaurants	Bars	Gyms	Stores	Mask mandate
New York	Jun 22	Aug 24	Jun 22	May 29	May 29	May 28
New Jersey	Jun 15	Sep 1	NA	Jul 8	Jun 15	Jun 8
Texas	May 1	May 1	May 22	May 6	May 1	NA
California	May 26	May 26	Jun 12	Jun 12	May 26	Jun 18
Florida	May 11	May 11	Jun 5	May 18	May 11	NA
Massachusetts	Jun 22	Jun 22	NA	Jul 6	Jun 8	May 18
Illinois	Jun 26	Jun 26	Jun 26	Jul 20	May 29	May 29
Pennsylvania	May 29	May 29	May 29	May 29	May 1	Jul 1
Michigan	May 18	May 18	May 22	Jun 10	May 26	Jul 14
Georgia	Apr 27	Apr 27	Jun 1	Apr 24	Apr 24	NA
Arizona	May 11	May 11	May 15	May 13	May 4	NA
Louisiana	May 15	May 15	Jun 15	May 15	May 15	Jul 11

**Figure 4:**
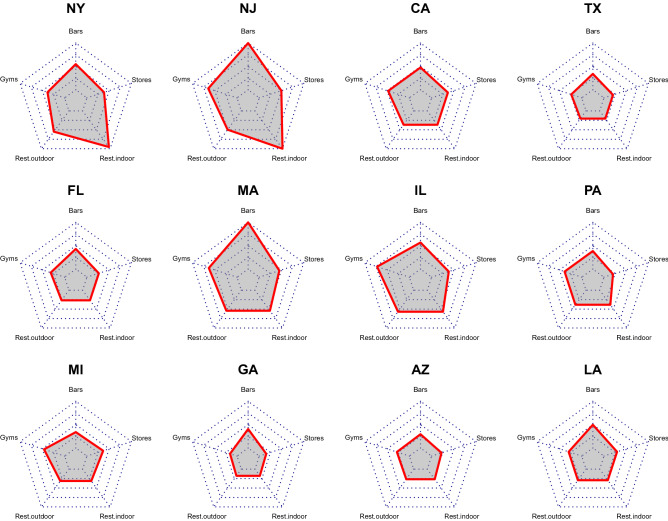
Radar charts for the reopening policies in different states. Five different categories are included: indoor restaurants, outdoor restaurants, gyms, bars, and stores. The earlier the reopening, the closer to the origin of the radar chart the vertex is. The zeroth day (origin of the radar chart) is April 1st, and the last day (the edge of the radar chart) is August 31st. A smaller pentagon implies a more aggressive approach in the reopening. The area does not necessarily imply a quantitative measure of the effect of reopening. It merely represents the dates of reopening of different categories of venues. Notes: The data can be found in Table 2.

For a quicker visual understanding of the reopening policies, we also plot the reopening dates of different categories as a radar chart for all the twelve states we consider (Fig. 4). The size of the pentagon is related to the speed of the reopening. The larger the pentagon, the slower the approach to reopening. By contrast, the smaller the pentagon, the more aggressive the reopening policies.

Previously, we investigated the effects of reopening by considering both the timing of reopening as well as the extent of the prevention measures implemented to lower the risk of infection after reopening. We found that lowering the infection rate is more important than the timing of reopening in the long term^76^. A probable way to lower the infection rate is by wearing personal protective gear, in particular a face mask^82^.

A simple indicator of the effect of different policies is the correlation between the given policy and the ratio between actual and projected death count, $R_{death}$. On this study, we focus on the reopening of five different venues and the mask mandate. A naive check of the validity of the correlation is that the reopening dates and mask mandate should have negative and positive correlation with $R_{death}$, respectively. An earlier reopening should cause higher death counts; conversely, enacting the mass mandate earlier should lower the death count. In addition, the magnitude of the correlation should represent the strength of the effect of each policy. If one particular category dominates, it indicates that particular category is the most important factor affecting the change in the death counts.

We list the Pearson correlation coefficients of each category in Table 3. The results are consistent with the expectation that the reopening dates have a negative correlation with $R_{death}$, and the date of enacting a mask mandate has a positive correlation with $R_{death}$. The Person coefficients show that the date of enacting the mask mandate has the strongest correlation with $R_{death}$. The measurement of the effect of different policies is based on the correlation between the change in the death counts and the reopening dates for different categories. The discussion below is based on this correlation.

**Table 3:** The Pearson correlation coefficients of six different categories of policies and the ratio between actual and projected death counts. The six categories are reopening of bars, gyms, outdoor restaurants, indoor restaurants, stores, and mask mandate.

Bars	Gyms	Restaurants outdoor	Restaurants indoor	Stores	Mask mandate
− 0.51	− 0.62	− 0.66	− 0.49	− 0.61	0.85

According to the data presented above, the four states (Texas, Florida, Arizona, Georgia) which have the largest increase of deaths due to reopening all have a common feature – a mask mandate has never been implemented. While the reopening date is markedly difference among different states, these four states tend to reopen early, but not by much compared to other states such as Michigan and Louisiana. The reopening dates do not seem to have as strong a correlation as the implementation of a mask mandate to the death count. This observation is supported by the largest magnitude (0.85) of the Pearson correlation coefficient for the mask mandate enacting date. For comparison, the Pearson correlation coefficients for the reopening dates of different venues fall between 0.49 and 0.66.

This observation seems to be consistent with the idea that the lowering of infection rate after reopening is more critical than the timing of reopening^75,76^. The reopening dates primarily affect the initial conditions of the epidemic, but the mask mandate affects the dynamics, in particular the infection rate, of the epidemic. There are five parameters in the model, $\beta , \eta , \alpha , \xi$, and $\delta$. $\beta$ is the infection rate, $\eta$ is the detection rate of infected patients, $\alpha$ is the recovery rate of asymptomatic infected people, $\xi$ is the recovery rate of the quarantined patients, and $\delta$ is the mortality rate of the quarantined. Among these five parameters, the mask mandate should only be able to explicitly affect the infection rate.

By the end of August, California and Louisiana did not show as substantial an increase in casualties as the above four states, although it is clear that the number of deaths per day is increasing starting around mid-July.

The two states with a lower death count than projected, Illinois and Massachusetts, both started mask mandates at earlier dates. Together with being comparatively late in reopening, they both show a clear decrease in death counts as one would predict from the trend in April.

While one can legitimately argue that correlation does not imply causation, it does seem that lack of a face mask mandate demonstrates the strongest correlation with the increase in the death count after reopening. Moreover, since different categories of policies were implemented in a convoluted timeline, it is difficult, if not practically impossible, to disentangle the contribution from each category without ambiguity.

While delaying the reopening should naively lower or at least delay the increase in the number of fatalities, it seems to be a secondary factor when comparing to the influence of the mask mandates. This observation is supported by the Pearson correlation coefficients presented in Table 3. One cannot directly identify the lack of a mask mandate as the cause of the increase in the number of casualties, as the states which do not have a mask mandate may also not have other policies which could help in lowering the infection rate. It is beyond the scope of the present study to uncover those possible causes.

## Discussion

The various mitigation efforts in the spring of 2020, in particular the stay-at-home orders, helped to stabilize the number of infections and casualties. All states passed or very nearly passed the phase of exponential growth in the number of infections by the end of April^1^.

Many other factors should have reduced the spread of the virus and the fatality rate. Awareness of the COVID-19 among the public and the general increasing trend of wearing face masks in public areas should contribute to the lowering of the infection count, and thus the death count. Together with substantial improvement in the number of tests administered as compared to last March and the reduction of the share of infections between senior citizens, all these factors should reduce the infection and the death counts. Thus, if the mitigation efforts had remained intact, the infection rate should have decreased over time. Therefore, the hypothetical projection presented in this paper represents an upper bound estimation, since it assumes these factors which potentially reduced the infection rate to be unchanged since the end of April.

The pressure to reopen starting in April led many state governors to relax their states’ mitigation efforts. The extent of the relaxation is different between states, as is the change in mortality^6^. Our analysis provides a quantitative assessment of the increase in the number of deaths due to the relaxation of the mitigation efforts and as consequence does or does not corroborate the states’ policies. This study should provide insight on what is a a good strategy for controlling the infection rate in the process of reopening. While various strategies do incur different impacts on human interactions and consequently the economy and many others, how to balance the effectiveness of lowering the infection rate against the negative effects of reducing human interactions is a broad topic which is beyond the scope of this study.

From the analysis presented in this paper, we found that three states show the largest increase in casualties due to their reopening policies: Texas, Florida, and Arizona. Georgia also shows substantial increase, though not to as great of an extent. On the other hand, Illinois and Massachusetts both show appreciable lowering of the death rate after April.

A common policy of the four states with the largest increase of deaths due to reopening is the absence of a mask mandate in the state. These four states tended to have reopened early, but not excessively so relative to other states. Therefore, we conclude that reopening dates do not seem to have correlate as strongly as the implementation of a mask mandate with an increasing number of infections and deaths.

It is an important issue to explore alternative hypotheses to explain the mismatch between the projected and the observed death counts. In the present study we attribute that to the reopening. An obvious alternative is the limitations of the model. As with most other SIR based models, there are many implicit assumptions. Our model is a coarse-grained mean field model which assumes the population is well mixed, and the interactions among the dynamical variables have no explicit time delay. The movement of population among different states and even countries is ignored in the present study. In the present study we treat each state as a closed system, no interaction outside the system of any kind is explicitly taken into consideration. For states which have frequent traffic flows, for example New York and New Jersey, an analysis of such interaction may be beneficial. It is also clear that the dynamics is different among different age groups^83^. There is also evidence which shows the racial and social economic disparities in the population infected by and dying from COVID-19^84^.

These are some of the examples for which their effects are not explicitly considered in the present model. A more sophisticated modelling should include all these effects into consideration, but given the limited data before the mitigation efforts were being relaxed, it would have been a rather challenging call to distillate those effects from all the others. Moreover fitting of the parameters is also a source of uncertainty. With the very limited set of data points before the relaxation of the mitigation efforts (about one month of data), it can be very misleading to provide “confidence interval” to our model with the usual meaning of say $95\%$ accuracy. Especially, there are evidences that at least some of the parameters are not self-averaging^2–5^. A more sophisticated model usually implies more parameters, and this problem would have been exacerbated. Undoubtedly, these are all possible sources of uncertainty which, at present, we do not have a good method to quantify them precisely.

A thorough survey of the differences between policies for states that show a marked increase in the death rate compared to those that show a reduction should provide guidance for a strategy which achieves the best results for lowering the number of infections and deaths.

In reality, relaxing the mitigation efforts is perhaps not purely a scientific problem, as it involves economic, political, and other social considerations. A delicate balance between all of those factors may need to be found.

Given that the winter season may present another wave of infections, a thorough study is particularly time sensitive. Our analysis seems to suggest that the face mask mandate is the most important policy for lowering the death count. Finally, we note that seasonal effects are not considered in the present study^85–87^.

The datasets generated during and/or analysed during the current study are available from the corresponding author on reasonable request.

## Appendix

We discuss the method of obtaining the initial condition and the parameters of the model in this appendix. At the beginning, very small fraction of the population is infected, assume the susceptible population count is the same as the total population, $S \sim N$. With this assumption, we obtain

9 $$\frac{dI(t)}{dt} = \beta \frac{S(t)I(t)}{N} -(\alpha +\eta )I(t) \approx [\beta -(\alpha +\eta )]I(t).$$

Solving the equation for *I*(*t*):^12,14^

10 $$I(t) \approx I(0)\exp [(\beta -(\alpha +\eta ))t],$$

At the very early stage, $Q \ll I$, we can solve for *Q*(*t*),

11 $$\frac{dQ(t)}{dt} = \eta I(t)-\delta (t) Q(t) - \xi (t) Q(t) \approx \eta I(t).$$

With the above solution for *Q*(*t*), we obtain

12 $$Q(t) = \frac{\eta }{\beta -(\alpha +\eta )}I(t).$$

We can then relate the rate of increase in the number of casualties with the number of infected people in the early stage of the epidemic:

13 $$\displaystyle \frac{dC(t)}{dt} = \delta (t) \frac{\eta }{\beta -(\alpha +\eta )} I(t) = \delta _0 I(t),$$

Finally, the casualty count can be found,

14 $$C(t) = \frac{\delta _0 I(0)}{\beta -(\alpha +\eta )}\exp [(\beta - (\alpha + \eta ))t].$$

In the early stage of the epidemic, we assume exponential growth in the number of fatalities before the mitigation efforts are implemented. We can find the initial exponent, $\beta -(\alpha + \eta )$, and its coefficient, the number of deaths per day are fit to Eq. (14) and its derivative $\frac{dC(t)}{dt}$, with the first date with one death taken as $t=0$.

Data is smoothed by preforming a three day moving average before using for fitting.. We discard data with less than ten deaths. To identify the initial number of infected people, *I*(0), we need to estimate $\delta _0$.

We assume the accumulated mortality rate is estimated to be 2.3%^78^. Notably, the mortality rate does indeed vary by region. Notwithstanding these uncertainties, the median time between infection and the onset of symptoms is about five days^2–5^. The median time between the onset of symptoms and death is about eight days^2–5^. With these information, we can estimate $\displaystyle \delta _0 \approx \frac{0.023}{5+8} \approx 0.0018/$day.

The average time to recovery or death from infection are both 13 days and that half of the infected never show any symptoms^79^. We estimate $\alpha =0.5/13 \approx 0.0385/$day.

We estimate $\eta$ by minimizing the $\chi ^2$ of the total number of deaths and confirmed cases as well as their derivatives. With $\eta$, we can deduce the infection rate $\beta$ and the reproduction number $R_0 \approx \beta /(\eta +\alpha )$^14^.

We also estimate both the death rate, $\delta (t)$, and the recovery rate of the quarantined, $\xi (t)$, from the raw data of confirmed cases and deaths as a function of time. With the assumption that the average time from the onset of symptoms to death or recovery is eight days, we obtain $\delta (t)+\xi (t)=1/8=0.125$/day. We can find $\delta (t)$

15 $$\delta (t) \approx \frac{1}{8}\frac{D(t+4)-D(t-4)}{P(t-4)-P(t-12)-D(t-4)}.$$

With $\delta (r)$, $\xi (t)$ can be found using the relation $\delta (t)+\xi (t)=1/8=0.125$/day^75^.

We list the parameters of the model obtained after the stay-at-home order was enacted and became effective in the Table 4.

**Table 4:** Table for the parameters of the model. $\beta$ is the infection rate, $\eta$ is the detection rate of infected patients, $\alpha$ is the recovery rate of asymptomatic infected people, $\xi$ is the recovery rate of the quarantined patients, and $\delta$ is the casualty rate of the quarantined. All of the parameters are in units of (1/day).

State	$\beta$	$\eta$	$\alpha$	$\xi$	$\delta$
New York	0.0580	0.0700	0.0385	0.1156	0.0094
New Jersey	0.0827	0.0910	0.0385	0.1134	0.0116
California	0.0978	0.0700	0.0385	0.1169	0.0081
Texas	0.1160	0.1010	0.0385	0.1200	0.0050
Florida	0.0762	0.0910	0.0385	0.1178	0.0072
Massachusetts	0.1429	0.1190	0.0385	0.1110	0.0140
Illinois	0.1469	0.1060	0.0385	0.1151	0.0099
Pennsylvania	0.1299	0.1110	0.0385	0.1147	0.0103
Michigan	0.0358	0.0570	0.0385	0.0978	0.0272
Georgia	0.0945	0.0320	0.0385	0.1187	0.0063
Arizona	0.1032	0.0820	0.0385	0.1163	0.0087
Louisiana	0.0190	0.0400	0.0385	0.1042	0.0208
